# Symmetry Restoring Bifurcation in Collective Decision-Making

**DOI:** 10.1371/journal.pcbi.1003960

**Published:** 2014-12-18

**Authors:** Natalia Zabzina, Audrey Dussutour, Richard P. Mann, David J. T. Sumpter, Stamatios C. Nicolis

**Affiliations:** 1 Mathematics Department, Uppsala University, Uppsala, Sweden; 2 Research Center on Animal Cognition, Université Paul Sabatier, Toulouse, France; University of Pittsburgh, United States of America

## Abstract

How social groups and organisms decide between alternative feeding sites or shelters has been extensively studied both experimentally and theoretically. One key result is the existence of a symmetry-breaking bifurcation at a critical system size, where there is a switch from evenly distributed exploitation of all options to a focussed exploitation of just one. Here we present a decision-making model in which symmetry-breaking is followed by a symmetry restoring bifurcation, whereby very large systems return to an even distribution of exploitation amongst options. The model assumes local positive feedback, coupled with a negative feedback regulating the flow toward the feeding sites. We show that the model is consistent with three different strains of the slime mold *Physarum polycephalum*, choosing between two feeding sites. We argue that this combination of feedbacks could allow collective foraging organisms to react flexibly in a dynamic environment.

## Author Summary

Collective decision making is ubiquitous in group-living organisms allowing them to select between several competing resources. It is a self-organized process involving positive feedback mechanisms, whereby the preference for a particular option is reinforced if the option has already been accepted by a part of the group's constituting units. The generally accepted paradigm of collective decision-making is a transition from an exploitation mode where all options are on equal footing, to one in which groups of sufficiently large size are led to focus on a particular option, a phenomenon referred to as symmetry-breaking bifurcation. In the present work we report results based on mathematical modeling in parallel with experiments carried out on the unicellular plasmoidal organism *Physarum polycephalum* showing that, contrary to the classical paradigm, symmetry is eventually restored for individuals of sufficiently large size (here the plasmodium mass). This possibility, arising from the combination of positive feedbacks and a regulation of the flow by the fraction of system's mass already committed to the options, allows the organism to react flexibly. We argue that, beyond the case of *P. polycephalum*, this paradigm should apply to many systems possessing the aforementioned feedback and regulatory mechanisms.

## Introduction

Many social or gregarious living organisms are effective decision-makers, in the sense that they are able to select the best of several available options [1]–[9]. Extensive experimental work and mathematical modelling suggest that a basic feature underlying this phenomenon is a symmetry-breaking bifurcation. That is, there is a transition from a “homogeneous” exploitation of the resources (all options are equally exploited) to an “inhomogeneous” mode where a focus on a particular option is occurring after a certain critical value of a parameter, typically the number of individuals [3], [10]–[13]. A key factor in the emergence of such patterns of exploitation is the amplification of an initial asymmetry arising through a fluctuation. For example in social insects, an individual discovering a food source will produce a signal that will be followed and reinforced by recruited individuals [14]. If the number of individuals is large enough, a slight initial imbalance of the fraction of individuals visiting one or the other source will entrain the majority of foragers to focus on a particular food source resulting in a collective decision. Such collective decision-making has been seen in predator avoidance [15], shelter selection [16] and has even been interpreted in terms of rationality [17], [18]. The idea of a symmetry-breaking depending on the number of individuals have also inspired other fields of research focusing on human behaviour [19]–[21] or economics [22], [23]. In all these examples, symmetry is broken when a critical number of individuals is exceeded.

While symmetry breaking is important, we also know that symmetry can be restored when the system size (e.g. number of individuals) becomes very large. For example, direct contacts resulting from crowding in foraging ants lead to the exploitation of two routes to food, despite the fact that only one route is chosen when there is no crowding [24]. More intricate situations can arise in, for example, ant species using two pheromones [25] or in social caterpillars *Malacosoma disstria* displaying behavioural polymorphism [26], [27]. Here exploitation patterns are shown to arise in which past a first symmetry-breaking transition there is coexistence of inhomogeneous and homogeneous modes the latter becoming even the rule under certain conditions. The interplay between symmetry breaking and symmetry restoring is also a basic issue in statistical and condensed matter physics [28], [29] and in high energy physics [30] when more than two phases of matter can coexist.

In this paper, we analyse decision-making at the cellular level, on the paradigmatic case of the true slime mold *Physarum polycephalum*. We show that non-trivial decision patterns, including a symmetry restoring bifurcation may arise depending on the mass of the slime mold.

**Figure 1:**
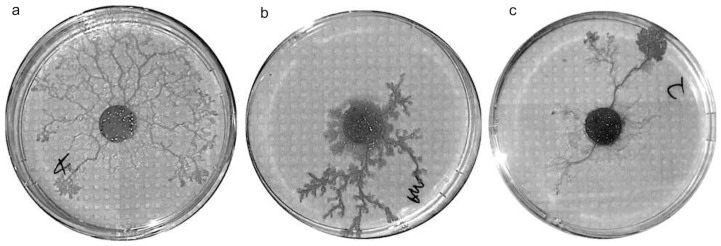
Exploration patterns of the three different strains tested. (a) Australian, (b) American and (c) Japanese.

*P. polycephalum* is a unicellular, multinucleate protist. Its vegetative phase is a multi-nucleate plasmodium. It is during this stage that the organism searches for food. Depending on the strains of the organism considered, the plasmodium sets out pseudopodia in all directions for a certain distance and then builds one or few extended search fronts (Fig. 1) during exploration. The plasmodium is able to sense various stimuli from a distance and move toward them via chemotaxis [31]. When the plasmodium comes into contact with a food source, it completely surrounds it and resumes exploration while remaining in physical contact with the initial food source. The plasmodium can grow to cover large area (up to 900 cm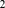), and is capable of moving at relative high speed (up to 5 cm/hr) [32] and of building efficient transportation networks [33]. In most of these studies reported in the literature, a single strain was used to reveal the decision-making patterns of *P. polycephalum*. In this paper we develop a model based on [34] and [35], along with experiments carried out on three strains of the true slime mould *P. polycephalum* to test our predictions and reveal the differences between these three strains in decision-making outcome.

The model describes how commitment to two identical options evolves in time. We let 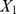 and 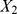 be the number of units within the system committed to options 1 and 2, respectively. We further assume a pool of uncommitted units of size 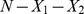 where 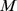 is the system size. We express the build up of commitment to the options with respect to time as 
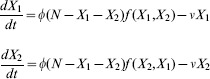
(1)


Here 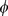 is the rate per individual unit time to choose between one of the options, 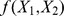 accounts for the feedbacks present in the decision-making and 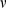 is the rate at which commitment decays.

A number of authors [1], [13], [24] have analysed a similar model – particularly in the context of foraging in ants – under the hypothesis that rate of decision-making is constant, i.e. 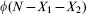 is replaced by a constant 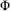. This hypothesis is reasonable in the limit where the initial system size is large and the number of units committed to the options remains small. However, in many natural systems the initial mass is significantly depleted as time goes on. This is certainly the case in our current experiment on foraging by *P. polycephalum* where a substantial part of the initial mass ends up covering one or both the food sources. The system we study here can thus be viewed as having a “passive” negative feedback of 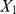, 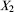, whereby depletion of units reduces the rate of recruitment.

We turn next to the positive feedback functions 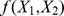. Several possible forms have been proposed for these (see [35] and [36] for recent reviews). One of the dominant ideas has been that an individual bases its decision on previous decisions made by others, i.e., on the numbers of units having already committed the different options. For example, [13] use 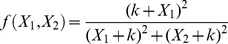
(2a)while, [37] argue, on the basis of Bayesian estimation, that the form 
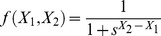
(2b)gives a form of optimal decision-making, 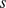 being a sensitivity parameter.

Both the above forms assume that information about commitment to both of the options is available to the decision-making units. An alternative view is the quorum model [34]. Here one assumes that the probability of accepting an option is simply an increasing function of the number of units that have already accepted this particular option independent of the number of units choosing the other option. In this paper we model this phenomenon using 
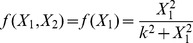
(2c)


This form follows [34], albeit without an additional spontaneous probability of adopting an option. A similar form has also been derived by [36] within a Bayesian framework. They found that 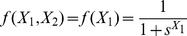
(2d)provided a good match to decisions made by zebrafish. As it turns out the use of either (2c) or (2d) is not essential in what follows. Both these functions have the same sigmoidal form, which produces the sequence of bifurcations we now describe.

In the case of *P. polycephalum* feedback is in the form of the growth of tubes as a result of protoplasmic flow. There is evidence that there is an upper limit of tube thickness in real organisms [38]. The parameter 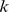 then stands for the threshold beyond which this feedback becomes effective or, alternatively the threshold flow for tube construction. Nakagaki et al. [39] consider the sigmoidal function of the form 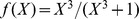 to account for these effects but, again, the results reported below are not affected qualitatively by the choice of exponents greater than 2.

Summarising, model (1) for two equal food sources can be written as
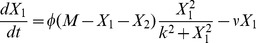


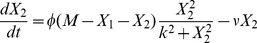
(3)


It captures two essential properties of a class of decision-making systems of which *Physarum polycephalum* constitutes a prototypical example. First, decisions are local in the sense that each of the two positive feedback functions 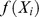 depends only on the fraction of system's mass attracted to the particular option 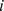. In particular, in *P. polycephalum* tubes are being built to food sources on the basis of only local information. Second, for any given value of initial mass 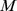 the portion of the system not yet committed to the options is decreasing as 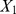, 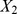 are increasing.

In order to investigate the role of randomness in the model and to fit it to data, we also implemented a Monte Carlo version of this model. See Materials and Methods for details.

## Results

**Figure 2:**
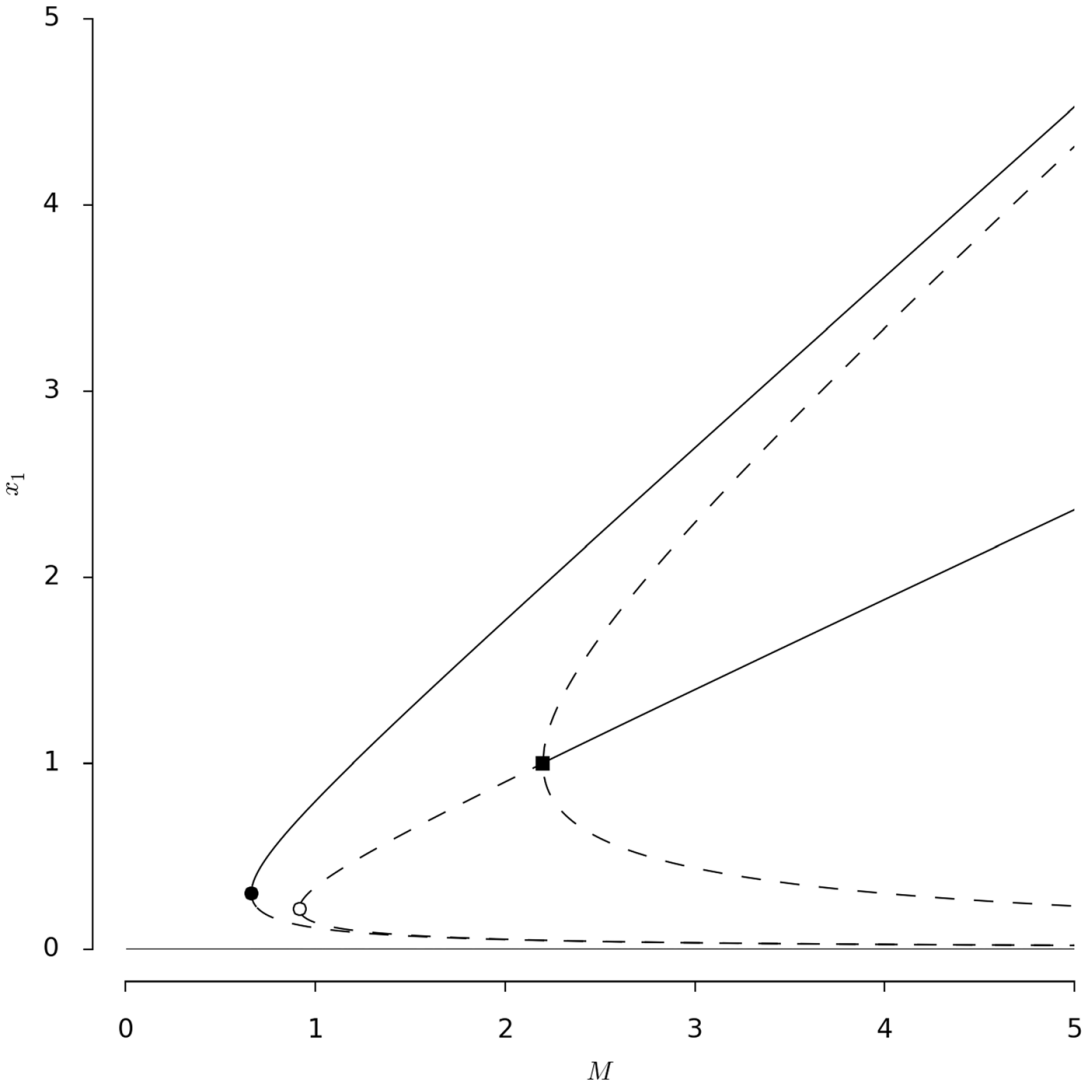
Bifurcation diagram corresponding to the steady state solutions of **equation (3**) with respect to the parameter 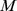. Full and dashed lines correspond to stable and unstable solutions respectively. The black circle shows the first bifurcation, the white circle corresponds to the second bifurcation and the black square labels the third bifurcation. Parameter values are 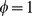, 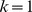 and 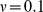.

Fig. 2 shows the bifurcation diagram of the steady-state solutions of eqs. (3), i.e., how the steady-sate level of commitment to an option changes for initial system sizes. Three bifurcation points can be identified. Before the first bifurcation there is one stable steady state corresponding to no decision (trivial steady state). After the first bifurcation point (see Material and Methods, eq. (6)) the system has three stable states, one corresponding to no decision and the other two corresponding to the exclusive exploitation of one or the other of the two options (semi-trivial steady state). In terms of the behavior of *Physarum polycephalum*, the trivial steady state describes a situation where the plasmodium did not find food or never moved from the starting point. The semi-trivial steady state describes the situation where the plasmodium exploits just one option.

For larger initial mass values a second bifurcation occurs and unstable homogeneous solutions appear. In terms of the decision-making of *Physarum polycephalum* the instability of these symmetric solutions means that the plasmodium does not have enough mass to exploit two options at the same time and thus moves to just one. After a critical value 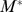 (see Materials and Methods, eq. (10)), corresponding to a third bifurcation, the upper branch of the homogeneous solutions becomes stable. This corresponds to the plasmodium equally exploiting both food sources. We label the bifurcation at 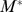 a symmetry restoring bifurcation, since a stable, nontrivial symmetric solution appears at this point.

This stabilisation coincides with the appearance of two non-homogeneous (asymmetric) unstable solutions, characteristic of a subcritical pitchfork bifurcation. Here we have tristability such that, depending on initial conditions, the plasmodium will exploit either none of the options, one of the two or both. The asymptotic analysis of these solutions for 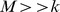 shows that the distance between the inhomogeneous solutions and the stable upper branch of the semi-trivial steady state decreases as 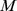 increases. This means that for large mass these two solutions are approximately equal. As a result, the stable upper branch of the semi-trivial solution(see Material and Methods, eq. (5)) can never be reached in the sense that the set of initial conditions in its attraction basin decreases in size with 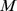. The biological conclusion is that a plasmodium of very large mass nearly always spreads between two options rather than moving to one.

**Figure 3:**
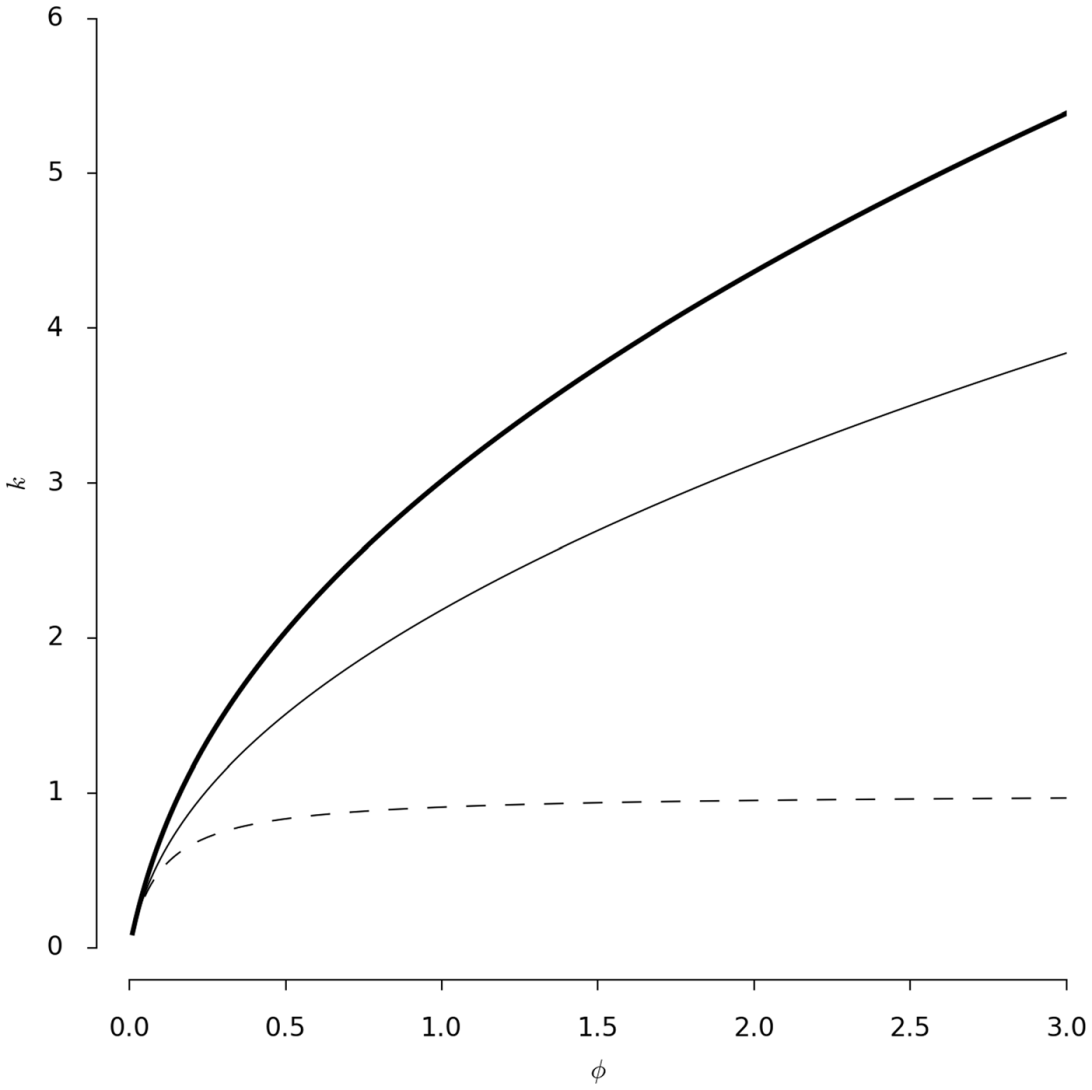
Conditions for existence of the bifurcation points displayed in **Fig. 2.** Parameter 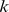 as a function of 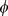 for fixed mass 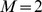, other parameter as in Fig. 2. Bold solid, solid and dashed lines correspond to the condition for existence of the first, second and third bifurcation, respectively.

We now study the role of the threshold and flux parameters 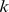 and 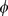. Fig. 3 depicts critical values of parameter 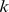 as function of parameter 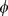 for the fixed mass 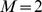. The three lines correspond to the three types of bifurcations identified above. The bold solid line corresponds to the condition of the first bifurcation to occur and thus, to the existence of semi trivial solutions (see eq.(6) in Materials and Methods). The solid line corresponds to the condition of the second bifurcation to occur and to existence of a non-trivial unstable homogeneous solution (see eq. (8) in Material and Methods) Finally, if parameters 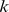 and 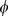 are chosen under the dashed line in Fig. 3 the existence of all types solutions and all bifurcation points is secured.

**Figure 4:**
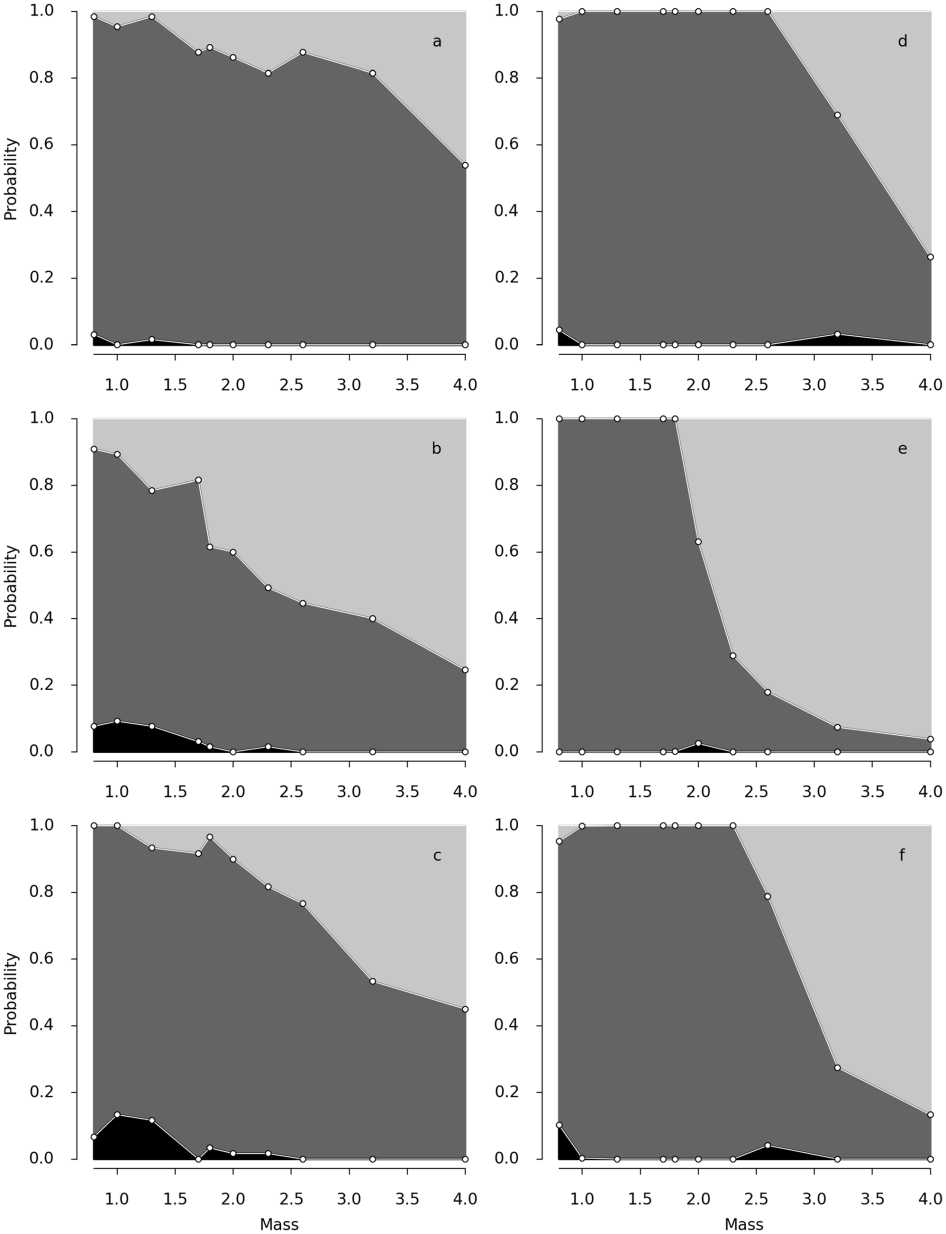
Probability to choose options with respect to the mass of plasmodium. The grey colour corresponds to the probability to move to the one option, the light grey shows the probability to move to two options at the same time and the black colour corresponds to probability to exploit zero option. Experiment outcomes a) Japanese strain, b) Australia strain and c) American strain. Model outcomes d) Japanese strain 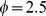, 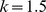, e) Australian strain 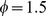, 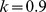 and f) American strain 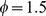, 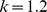, other parameter as in Fig. 2.

We next turn to the experimental results. Fig. 4a,b,c shows the probability to move to a food source as a function of the size of plasmodium. For very small size, there is a non-negligible probability to select none of the food sources. Plasmodia of small masses exploit more often only one source, while larger ones exploit both food sources at the same time. For example, the smallest Japanese plasmodia (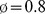 cm) exploit only one food in 95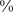 of the cases while for the largest size (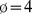 cm), this frequency decreases to 54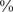 (see Fig. 4a). Similar results are observed for the other strains (see Fig. 4b, c). We notice however that there are some quantitative differences of exploitation patterns between strains: The largest Australian plasmodia (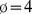 cm) exploit two food sources in 75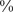 of the cases, a value which is larger than for the two other strains. Decision making by *Physarum polycephalum* depends thus on the size of the plasmodium as well as on the different exploration patterns of the strains.

The experimental results are qualitatively consistent with the model predictions. Indeed, for small values of the parameter 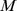, there is no option chosen. For larger 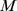, there is coexistence between a state where one option is chosen and a state where no option is selected. Finally, for still larger 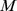, there is coexistence between three states corresponding to the selection of one option, to the simultaneous selection of two options and no selection at all. In terms of *Physarum polycephalum*, an organism with a small mass exploits one or no options, while a large mass endows it with the possibility to select simultaneously two options, one option or none.

In order to compare the predictions of the model to the experimental outcome, we identified the best fit model in terms of the parameters 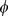 and 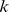 for each strain. For each mass 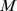 used in the experiment we performed a Monte Carlo simulation of the model (Materials and Methods) for different parameter combinations. We run the Monte Carlo simulation 1000 times for each pair of 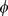 ranging with the step 0.1 from 0.5 to 3.5 and 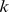 ranging from 0.5 to 2, and identified the best fit parameters (see eq. (12) in Materials and Methods for details of model fitting).

The best-fit parameters identified for each strain of plasmodium are given in Table 1, along with the goodness of fit parameter (see Model fitting in Materials and Methods section). The fitting parameter 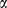 can be roughly interpreted as the proportion of data explained by our Monte Carlo simulation model. It varies for each strain between 0.84 and 0.92, indicating that the simulation model accounts for the large majority of observed variation, supporting the validity of the inferred values of 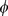 and 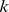.

**Table 1:** Best-fit parameters obtained from eq. (12).

strains	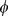	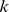	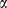
Japan	2.5	1.5	0.9239
Australian	1.5	0.9	0.8448
American	1.5	1.2	0.9149

Fig. 4d,e,f shows the probability of selecting an option as a function of the mass, resulting from an average of 1000 realisations for every value of the mass considered and from the best fit parameters shown in Table 1. This is to be compared with the experimental probabilities (Fig. 4a,b,c). The model captures adequately the different patterns of exploitation for the masses and the strains considered in the experiment.

**Figure 5:**
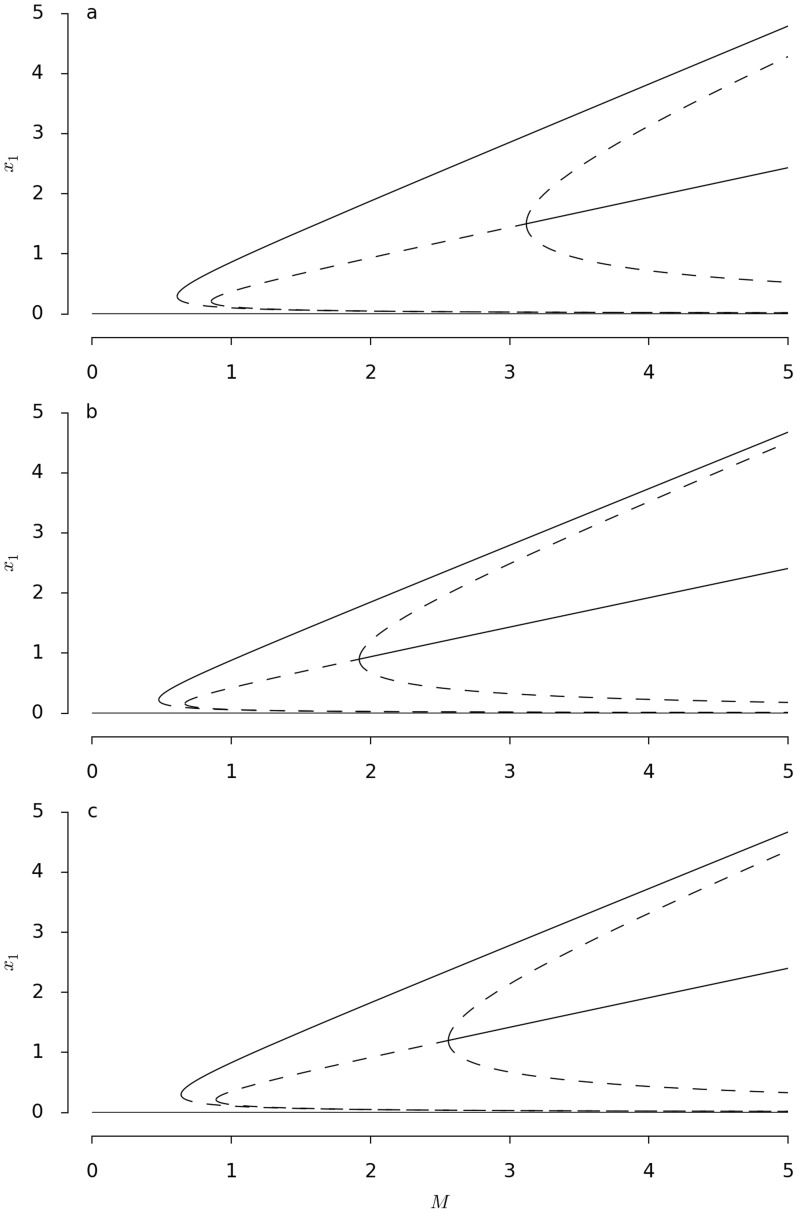
Bifurcation diagrams corresponding to the steady state solutions of **equation (3)** with respect to the parameter 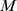 corresponding to the three different strains. Full and dashed lines correspond to stable and unstable solutions respectively. Parameter values are a) Japan 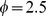, 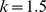, b) Australian 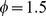, 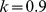 and c) American 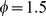, 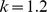, other parameter as in Fig. 2.

Fig. 5 shows the bifurcation diagrams corresponding to the best-fit parameters for each of the three strains. We now identify the positions of the symmetry-restoring bifurcation point beyond which a simultaneous exploitation of the two options becomes possible. We notice that the critical value of the mass 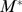 is different for the three strains, the Japanese one occurring at 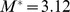 (cf. Fig. 5a) while the Australian and American ones occur at smaller values (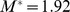 and 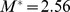 respectively, Fig. 5b,c).

These differences can be explained in biological terms and the exploration patterns of the slime mold (Fig. 1). The exploratory pattern of the Japanese strain is directional, forming thick tubes during its displacement. A larger mass is then needed to be able to exploit two options. In contrast, the Australian strain explores its environment more uniformly by forming thin tubes. A smaller mass is then needed to be able to exploit two options. As for the American strain, its exploration pattern combines both Japanese and Australian ones and an intermediate value of the mass is then needed.

These exploration pattern differences are taken into account by the differences between two parameters that we used in our model. 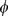 can be viewed as the speed of displacement of the plasmodium while 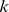 reflects a threshold beyond which a tube can be built, and therefore is related to the way the different strains are moving: a small value of 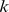 means that a tube is more easily constructed, even with a low mass. The function 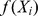 in eq. (4a) saturates therefore more quickly and favours the homogeneous solution. On the contrary, a large value of this parameter implies that a large mass will be needed to build a tube and that 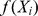 saturates more slowly, favouring the semi-trivial inhomogeneous solution (6).

## Discussion

We have presented a generic mathematical model for how different patterns of exploitation of two identical resources depend on the size of the system. The model takes into account two important features. Firstly, owing to the finite size of the system, the number of uncommitted units is limited by that already committed to the feeding sites. Secondly, the amplification process is local in that no direct comparison is made between the two options. The combination between local positive feedback and regulation of the traffic revealed a symmetry restoring bifurcation beyond which the system was able to select simultaneously two options, one of two options, or none of them. Past this bifurcation point, for increasingly larger initial system sizes, this tristability was still present but the symmetric solutions had an increasing basin of attraction. This was due to the existence of nearby unstable inhomogeneous states masking the other stable states.

Most of the studies investigating decision-making patterns in *Physarum polycephalum* were conducted using a single strain (the Australian strain obtained from Southern Biological Supplies: [40]–[42]; the Japanese strain: [43]). In order to test the model, we conducted experiments on three strains of *Physarum polycephalum*, each of them having different pattern of exploration. In our experimental set-up we took single individuals of different masses and let them choose between two identical food sources on a Petri dish. The different types of exploitation patterns obtained were similar to those predicted by the model, with the model capturing around 90% of the data.

Symmetry-restoring is a generic phenomenon resulting from the coexistence of positive and negative (regulatory) feedbacks. In addition to the case considered in this work, it is also encountered in social insect foraging [24], [25]. Beyond the case of decision-making in biological organisms, symmetry restoring is known to be also present in physical sciences, including phase transitions [29] and pattern formation in reaction-diffusion systems [44].

Our study highlights an important difference between local and global information in decision making. In slime mould, flow is a function of the thickness of the tube between the organism and a specific food source [38], [39]. As a result, tube growth is a local process in the sense that tubes oriented along different directions are not inhibiting growth. In many experiments on ant and fish decision-making there is a predetermined decision point, at for example a Y-shaped branch [3], [15], where animals compare the two options directly. This choice point provides global information. It would be interesting to investigate situations in ants and other social organisms in a natural environment where groups are still offered two options (two food sources) but there is no pre-determined choice point. A setup of this kind for ants could consist of colonies of variable sizes connected to an open arena containing two identical food sources placed equidistant to the nest. The traffic that will eventually be established will still privilege paths leading to the food sources, but the information held by individuals will be purely local. In these conditions, we predict that beyond a critical size of the colony, individuals will display the three exploitation patterns seen in this paper. In particular, we predict a symmetry restoration at large colony sizes. Notice that symmetry restoring should be possible even in a maze type experiment provided that returns to the main branch of the maze can occur. A full analysis of this problem would require to incorporate in the description the navigation strategies employed. This is beyond the scope of the present work.

The coexistence of multiple steady states in our model is expected to enhance flexibility. In nature, food sources are not constantly available and colonies focussing on one source can take a long time to switch to another [45]. However, in the region of coexistence between many solutions, a colony may quickly switch to another option [35]. Previously this was shown to be the case in the presence of crowding [24] or in the presence of more than two options [18]. We suggest that this may also happen in an open environment in the presence of only two options and a regulation of traffic of the kind considered in this paper.

## Materials and Methods

We studied the model presented in the Introduction in two ways, as a system of coupled differential equations as defined by eqs. (3) and via a Monte Carlo simulation.

### Steady states and stability

We start by studying steady-state (time-independent) solutions of the system (3). Setting time derivatives to zero and denoting by 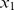 and 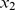 the steady state solutions we arrive at the following system of algebraic equations
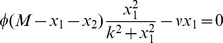


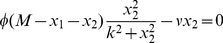
(4)


By solving this system we can determine how the decision to choose one, two or zero options depends on the total mass 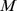. We notice that eqs. (3)–(4) secure positivity of 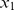, 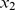 as well as the property 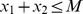 whatever the values of 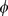, 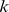 and 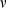 might be, provided that these conditions are satisfied initially. Indeed, as 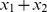 approaches 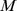 starting from smaller values the first (positive) term in the rhs of eq. (4) will become increasingly small and the second (negative) term will dominate. As a result the time derivatives in eq. (3) will be negative and 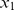, 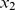 and their sum will be led to lesser values.

By evaluating the Jacobian at the steady states we can also determine their stability. In the general case, the Jacobian is
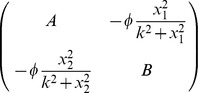
where 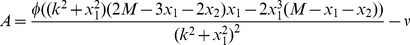
and 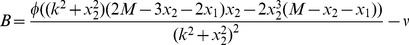



Thus the characteristic equation determining the eigenvalues of the Jacobian has the form 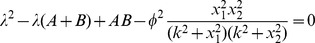



The steady states are stable as long as the real parts of the two (possibly complex) eigenvalues are negative. Equations (3) admit four types of steady states. We now discuss the existence and stability of each of these in turn.

#### The trivial solution 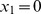 and 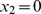


This solution is always stable with corresponding double negative eigenvalue 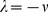.

#### The semi-trivial solutions 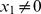 and 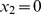


To find these solutions we let 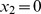 in equations (3) then by simplifying we get 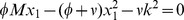



This gives 
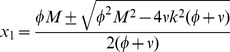
(5)


Among the solutions of the system (3) only real, positive solutions are acceptable. For the semi-trivial solution (5) to be real and positive we thus need 
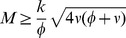
(6)


The equality sign gives the critical mass at which a limit point bifurcation occurs, see Fig. 2. Substituting the values of 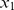, 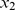 in the characteristic equation one finds that the semi-trivial solution corresponding to the upper branch of 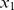 is always stable and the lower branch is always unstable.

For large 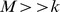 the upper branch is 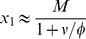
while the lower branch tends to 0 as 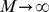.

#### The non-trivial homogeneous solutions 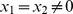


To find the solutions we set 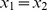 in the first equation of the system (3). By simplifying we get
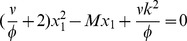
so that 
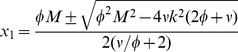
(7)


These solutions are real and positive if 
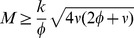
(8)


The equality sign gives the critical mass at which a second limit point bifurcation occurs, see Fig. 2. Substituting 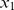, 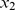 in the characteristic equation one sees that these states are unstable for small values of 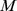. But as 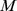 is increased well beyond 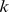, it turns out that the solution becomes stable and tends for large 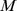 to 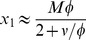
while the lower branch is unstable and tends to 0 as 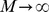.

#### The fully non-trivial and non-homogeneous solutions 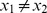


To find these solutions we have to express 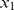 from one equation of the system (3) in terms of 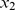 and substitute. The calculation reveals 6 solutions, but 4 of these are already described above. The remaining two solutions are 
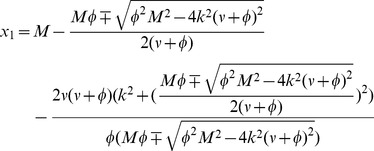
(9)


These solutions are real and positive as long as 
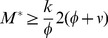
(10)


The equality sign gives the critical mass at which solutions (9) are merging with the homogeneous branch (7). While these solutions are always unstable beyond the critical mass 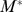, the unstable upper branch of solution (7) becomes stable.

The third bifurcation occurring at 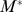 is thus a pitchfork type bifurcation. Notice that for 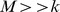 the upper branch of fully non-trivial solution behaves 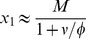
while the lower branch of 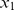 tends to 0 as 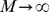. This is the same asymptotic result as for the semi-trivial solution (6).

Summarising, the model admits the following physically acceptable steady-state solutions: the trivial solution 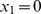 and 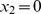 corresponds to the absence of a decision, the semi-trivial solutions 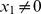 and 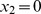 (or 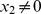 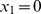) correspond to an exclusive exploitation of one option, the non-trivial homogeneous solutions 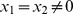 correspond to symmetric exploration of both options and the fully non-trivial and non-homogeneous solutions 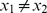 when 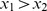 or 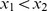 correspond to asymmetric exploitation.

### Monte Carlo simulation

In order to incorporate the fluctuations inherent to the experiments, we developed a Monte Carlo approach by simulating directly the equations (3). We describe the main principles of our Monte-Carlo simulation implementation in the following steps:

#### Initial condition

We assume that the number of the units within the system committed to option 1 and 2, 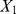 and 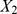 initially are not zero. These values are generated from the uniform distribution on interval 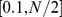, where 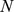 takes values of diameter values 4cm, 3.2 cm, 2.6 cm, 2.3 cm, 2 cm, 1.8 cm, 1.7 cm, 1.3 cm, 1 cm and 0.8 cm. Thus for different size of plasmodia initial values are generated from respective interval.

#### Decision process

The coupling in the choice between two options in our model (3) is weak because we use the choice functions which are independent to each other. The sum of coupled choice functions should be equal 1 thus they can be used as a probability to choose options. In our case first we have to define expressions: 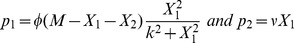


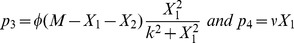
then we construct probabilities to move to the options in the following way: 
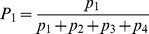


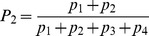


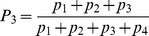


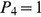
(11)


The decision concerns the movement of the mass to the options. To this end, a random number 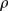 is sampled from a uniform distribution between 0 and 1:

- if 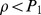, a small mass unit (taken here to be 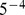) is added to 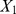 (while 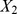 remains unchanged)
- if 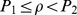, a small mass unit is removed from 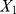 (while 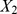 remains unchanged)
- if 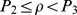, a small mass unit is added to 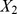 (while 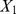 remains unchanged)
- if 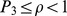, a small mass unit is removed from 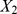 (while 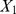 remains unchanged)

#### Time evolution

The probabilities represented by (11) are updated at each simulation step according to the actual mass movement to the particular option. The process is repeated for a number of steps (70 000) sufficient to reach the stationary state, where all presented mass 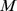 will move to the options, in another words when expression 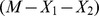 will be equal zero.

The simulations run for 1000 realisations and we calculate the average mass value on the options. If most (at least 60%) of the mass is found to have moved to a particular option, we conclude that one option has been selected. If the mass is found to have spread equally between the options, we conclude that two options have been selected. Finally, if most of the mass has not moved, we conclude that no option has been selected.

### Experiment

In the light of the model results, we conducted a series of experiments to determine how the mass of *Physarum polycephalum* plasmodium influences the foraging decision process when the individual is confronted with two identical food sources.

*Physarum polycephalum* is a unicellular, true slime mold, typically yellow in colour, and inhabits shady, cool, moist areas such as decaying leaves and logs. It belongs to the supergroup Amoebozoa. The main vegetative phase of *P. polycephalum* is the multi-nucleate plasmodium (the active, streaming form) that consists of networks of protoplasmic veins and pseudopods. It is during this stage that the organism searches for food. In the wild, the plasmodium eats bacteria and dead organic matter and in the laboratory they are fed oat flakes.

We cultivated *Physarum polycephalum* on a 10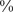 oat medium in a Petri dish (diameter: 145 mm). The rolled oat were grained and set in 1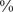 agar solution for presentation to *Physarum polycephalum*. To compare the foraging solution predicted by the model with those of *P. Polycephalum*, we measured the foraging solutions produced by three different strains: Australian strain (Southern Biological Supplies, Victoria), American strain (Carolina Biological Supplies) and from Japanese strain (Strain HU192 x HU200) that exhibit different exploration patterns. The Japanese strain is fast, forming only a few thick tubes to explore the substrate covering a long distance but a small surface. The Australian strain spreads in all direction by forming multiple thin tubes, covering a large surface but a small distance. The American strain combines both exploration patterns. It forms both thick and thin tubes (see Fig. 1 for a snapshot of the three different exploration patterns by these three strains).

In each trial one single plasmodium was confronted with two identical food sources. The food consisted in a 10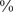 oatmeal-agar mixture similar to the one used for rearing the plasmodia. The foraging arena were made by filling 90-mm diameter Petri dishes with plain 1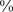 agar. Once the agar set, we punched two circular holes (diameter: 1.7 cm, 2.5 cm away from each other) into the agar and filled them with food. Then we punched a third circular hole placed 2.5 cm away from each source which we filled with a plasmodium. The diameter of that last hole varied depending on plasmodium size.

We tested 10 plasmodium sizes (by extension, 10 plasmodium masses) corresponding to the following diameters: 4 cm, 3.2 cm, 2.6 cm, 2.3 cm, 2 cm, 1.8 cm, 1.7 cm, 1.3 cm, 1 cm and 0.8 cm. The distance between the border of the plasmodium and the food was kept at a fixed value equal to 2 cm, whatever the diameter tested.

We replicate each experiment 65 times for each plasmodium size and each strain (1950 experiments in total: 65 replicates 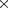 3 strains 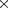 10 plasmodium sized). All the experiments were conducted in the dark at 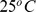 temperature and 70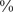 humidity. Experiments were run for 48 hours and pictures were taken every 5 min with a digital camera canon 60D.

Throughout the experiment the plasmodium explores its environment by deploying a network of protoplasmic tubes until a food source is discovered, whereupon a link between the food source and its initial position is built. We consider that a given source is chosen if the plasmodium moves toward it through the link and fully covers it. If on the other hand the plasmodium does not completely cover the food source and moves to the other one at the same time to eventually cover it in part, we consider that both sources are chosen. We recall that both experiment and theory concern the steady state behaviour. Transients are likely to be of interest as well, but are not addressed here. Finally, if after exploring the environment the plasmodium did not succeed in finding any food source during the time of experiment we consider that no choice has been made.

Summarising, we differentiated three distinct foraging patterns – the plasmodium exploits both food sources simultaneously, a single source and none of them – and calculated the proportion of replicates that ended up in these three states.

### Model fitting

We expect that our Monte Carlo simulation will capture a large proportion, but not all of the details of the real process. For example, for certain parameter values and masses our model predicts that the plasmodium will always move to exactly one option. In the data however, there is always some non-vanishing probability of a slime mould encountering two food sources. Acknowledging that our simulation model will not fully describe the many effects that could cause variation in the slime moulds behaviour, we must adapt our model fitting to allow for this in order to make our eventual fitted estimates of the simulation parameters robust. We thus modify our model fitting to account for variation that is not explained by the simulations, by fitting a mixture model comprising the simulation predictions, and a uniform distribution that represents all of the variation that is not accounted for in the simulation model. We thus introduce a new parameter, 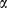, that controls the mixing proportion of the simulation predictions, and therefore represents the proportion of the experimental variation explained by the simulation model [46].

Mathematically, to do the fitting, we let 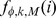 be the proportion of times the simulation with mass 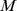 and parameters 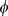 and 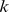 chooses 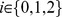 food options. We denote by 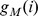 the experimental proportion of times a plasmodium of mass 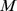 chose 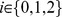 food sources. Let 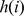 be this uniform distribution over the options, such that 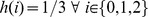. Introducing 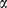 as the proportion of variation explained by our simulation and therefore the mixing ration of 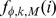, we have a prediction for the distribution of 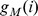: 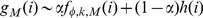
(12)where we infer 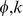 and 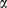 for each strain by finding the values that minimises the 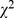 error term between this prediction and the experimental results. Identifying the best-fit values of the parameters is done by an exhaustive search over all combinations of 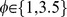, 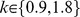 and 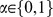 with steps of 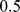, 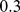 and 0.01 respectively. While the inferred parameters 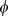 and 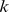 are the best estimates for the internal processes of the slime mould described above, the inferred value of 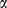 indicates what proportion of the experimental variation can be attributed to the processes specified in the simulation model, rather than to all other factors accounted for by the uniform distribution. It is therefore encouraging that the inferred values of 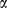 in our study are typically on the order of 0.9 (see Table 1). As seen, the large values of 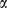 inferred indicate that our simulation predictions are a substantial improvement upon a null hypothesis that the slime mould chooses randomly between the three options.

## Supporting Information

S1 Text **The file contains the raw data of the experiments described in the Materials and Methods section.**
(XLSX)
